# Peptide binding prediction for the human class II MHC allele HLA-DP2: a molecular docking approach

**DOI:** 10.1186/1472-6807-11-32

**Published:** 2011-07-14

**Authors:** Atanas Patronov, Ivan Dimitrov, Darren R Flower, Irini Doytchinova

**Affiliations:** 1 Rebirth, Hannover Biomedical Research School, Carl-Neuberg st 1, 30625 Hannover, Germany; 2 School of Pharmacy, Medical University of Sofia, 2 Dunav st., 1000 Sofia, Bulgaria; 3 Life and Health Sciences, Aston University, Aston Triangle, Birmingham, B4 7ET, UK

## Abstract

**Background:**

MHC class II proteins bind oligopeptide fragments derived from proteolysis of pathogen antigens, presenting them at the cell surface for recognition by CD4+ T cells. Human MHC class II alleles are grouped into three loci: HLA-DP, HLA-DQ and HLA-DR. In contrast to HLA-DR and HLA-DQ, HLA-DP proteins have not been studied extensively, as they have been viewed as less important in immune responses than DRs and DQs. However, it is now known that HLA-DP alleles are associated with many autoimmune diseases. Quite recently, the X-ray structure of the HLA-DP2 molecule (DPA*0103, DPB1*0201) in complex with a self-peptide derived from the HLA-DR α-chain has been determined. In the present study, we applied a validated molecular docking protocol to a library of 247 modelled peptide-DP2 complexes, seeking to assess the contribution made by each of the 20 naturally occurred amino acids at each of the nine binding core peptide positions and the four flanking residues (two on both sides).

**Results:**

The free binding energies (FBEs) derived from the docking experiments were normalized on a position-dependent (npp) and on an overall basis (nap), and two docking score-based quantitative matrices (DS-QMs) were derived: QMnpp and QMnap. They reveal the amino acid preferences at each of the 13 positions considered in the study. Apart from the leading role of anchor positions p1 and p6, the binding to HLA-DP2 depends on the preferences at p2. No effect of the flanking residues was found on the peptide binding predictions to DP2, although all four of them show strong preferences for particular amino acids. The predictive ability of the DS-QMs was tested using a set of 457 known binders to HLA-DP2, originating from 24 proteins. The sensitivities of the predictions at five different thresholds (5%, 10%, 15%, 20% and 25%) were calculated and compared to the predictions made by the NetMHCII and IEDB servers. Analysis of the DS-QMs indicated an improvement in performance. Additionally, DS-QMs identified the binding cores of several known DP2 binders.

**Conclusions:**

The molecular docking protocol, as applied to a combinatorial library of peptides, models the peptide-HLA-DP2 protein interaction effectively, generating reliable predictions in a quantitative assessment. The method is structure-based and does not require extensive experimental sequence-based data. Thus, it is universal and can be applied to model any peptide - protein interaction.

## Background

Major histocompatibility complexes (MHCs) class II molecules are glycoproteins involved in the exogenous antigen processing pathway, responsible for presenting self and non-self peptides to inspection by T-cells. Class II MHCs are expressed on specialised cell types, including professional Antigen Presenting Cells (APCs), such as B cells, macrophages and dendritic cells. MHC class II proteins bind oligopeptide fragments derived through the proteolysis of pathogen antigens, and present them at the cell surface for recognition by CD4+ T cells. If sufficient quantities of the epitope are presented, the T cell may trigger an adaptive immune response specific for the pathogen. The peptides binding to MHC class II proteins vary considerably in length from 12-25 amino acids. They are bound by the protrusion of peptide side chains into cavities within the groove and through a series of hydrogen bonds formed between the main chain peptide atoms and the side chains atoms of the MHC molecule. The peptide is able to extend from either of the two open ends of the binding groove. It takes an extended polyproline-like conformation [1].

MHCs are the most polymorphic protein in higher vertebrates, with more than 6000 class I and class II MHC molecules listed in IMGT/HLA in February 2011 [2]. Determining the peptide binding specificities exhibited by this vast collection of alleles is beyond the present capacity of experimental techniques, necessitating the development of bioinformatic prediction methodologies. The most successful prediction methods for T-cell epitopes developed to date have been data-driven. T-cell epitope prediction typically involves defining the peptide binding specificity of specific class I or class II MHC alleles and then predicting epitopes *in silico*.

Using peptide sequence data, experimentally-determined affinity data has been used in the construction of many MHC-peptide binding prediction algorithms. Such methods include motif-based systems, Support Vector Machines (SVMs) [3,4], Hidden Markov Models (HMMs) [5-7], QSAR analysis [8,9], and structure-based approaches [10-12]. MHC binding motifs are an easily understood epitope identification method, although such motifs invariably generate numerous false positives and numerous false negatives.

At least for well-studied class I MHC alleles, immunoinformatic prediction methods work well [13,14]. However, for prediction of all immune epitope data other than class I MHC peptide binding, results have rarely proved satisfactory. Over the last few years, several comparative studies have shown that the prediction of class II T-cell epitopes is usually poor [15-17].

Human MHC class II alleles are grouped into three loci: HLA-DP, HLA-DQ and HLA-DR. Class II MHCs have been associated with many chronic inflammatory diseases [18], including rheumatoid arthritis and type 1 diabetes. Many crystal structures are now available for HLA-DQ and HLA-DR proteins [19], which show that the peptide binding site is composed of two separate chains: α and β. The walls of the binding site are formed by two anti-parallel helices and the floor is formed by an eight-stranded β-sheet [20]. Much of the extraordinary sequence polymorphism apparent in human MHCs is concentrated in residues forming the binding site. The site is open at both ends and peptides of different length could bind, even though only 9 amino acids occupy the site itself.

In contrast to HLA-DR and HLA-DQ, HLA-DP proteins have not been studied extensively, as they have been viewed as less important in immune responses than DRs and DQs. However, it is now known that HLA-DP proteins contribute to the risk of graft-versus-host (GVH) disease [21], sarcoidosis [22], juvenile chronic arthritis [23], Graves' disease [24], hard metal lung disease [25] and especially, chronic beryllium disease [26]. Quite recently, the X-ray structure of the HLA-DP2 (DPA*0103, DPB1*0201) in complex with a self-peptide derived from the HLA-DR α-chain has been determined [27]. Although the overall structure of DP2 is similar to that of other MHC class II proteins, it contains a unique solvent-exposed acidic pocket containing three glutamic acids (Glu^26β^, Glu^68β ^and Glu^69β^). This pocket may be able to bind Be and present it to T cells, thus explaining the mechanism of chronic Beryllium disease [27,28]. The X-ray data also revealed that the DP2 binding site consists of four binding pockets: deep and hydrophobic p1 and p6 pockets; large, shallow and negatively charged p4; and deep, narrow and polar p9.

Given the ready availability of the structural data to which we have briefly alluded above, the molecular docking has now become an appropriate tool, capable of application to the problem of binding prediction for class II MHCs. Structure-based docking is the repeated static docking - and subsequent empirical scoring - of sets of molecular structures to a biomacromolecular target, such as class II MHC complexes. The molecular docking has a burgeoning track-record of success, at least in area of identifying small molecule ligands of macromolecular targets, and can help identify MHC binders. Speaking generally, the molecular docking can be separated into five phases, beginning with the X-ray structure of a target MHC. This is combined with potential peptide binders. The resulting set of ligands is then docked into a binding site model and scored for some appropriate correlate of binding. Handful of the top ranked hits is selected, and assayed experimentally [29].

Specifically, in the present study, we applied a molecular docking protocol to a library of 247 modelled peptide-DP2 complexes to assess the contribution of each of the 20 naturally occurred amino acids at each of the nine binding core positions and the four flanking residues (two on both sides). The normalized binding scores formed a quantitative matrix (QM). The predictive ability of the QM was assessed by external test set of 457 known binders to DP2. A comparison with results generated by existing servers for DP2 binding prediction indicated an improvement in performance offered by our docking score-based QM (DS-QM).

## Methods

### Input data

The X-ray structure of the HLA-DP2 (DPA*0103, DPB1*0201) in complex with a self-peptide derived from the HLA-DR α-chain (pdb code: 3lqz) was used as a starting structure [27]. The covalently bound peptide was separated and defined as chain C. It consists of nine binding core positions (FHYLPFLPS) and six flanking residues (RK at the N terminus and TGGS at the C terminus). The conformation of the peptide was used as a template for the modelling process. Thirteen positions were examined: nine binding core positions and four flanking residues (two on both sides). A library of 248 peptides (19 amino acids × 13 positions + 1 original ligand) was built using PyMOL [30]. We used the SAAS (single amino acid substitution) approach to model the conformations of each altered side chains: after substitution, the side chain conformation was minimised while keeping the rest of the peptide structure and the whole MHC protein rigid. The protonation state of ionisable protein side chains was assigned to a standard ionisable state: neutral for His; positively charged for Arg and Lys; and negatively charged for Asp and Glu [31].

### AutoDock protocol

AutoDock 4.2 [32], employing an implementation of the Lamarckian genetic algorithm (GA), was used to model the peptide binding to HLA-DP2. In order to limit the computational burden of calculating peptide-MHC interactions at positions not involved in the static docking, we kept all coordinates fixed apart from the peptide residues of interest. These were left flexible. All GA settings were kept to their default values, apart from the number of energy evaluations and the number of generations which were set to 250 000 and 27 000, respectively. The docking grid was defined as a cuboid with sizes 32 Å × 36 Å × 38 Å, which encompassed the entire peptide binding site on DP2. The output from ten independent GA runs for each ligand was processed and the pose (binding conformation) with the lowest Free Binding Energy (FBE) was considered. FBE values represent the direct output from the AutoDock 4.2 scoring function which takes into consideration weighted terms for van der Waals dispersion/repulsion, hydrogen bonding, electrostatics, and desolvation interactions as well as the change in torsional free energy when the ligand goes from an unbound to bound state. Data was mined by python scripts using the MGL Tools 1.5.4 package [33]. All retained poses considered in the study had an RMSD below 1.5 Å.

### Docking score-based quantitative matrices (DS-QMs)

The FBEs derived from the docking experiments had negative and positive values. Negative FBEs correspond to binding peptides, while positive FBEs correspond to non-binding peptides. Only negative FBEs were considered; non-binding amino acids were assigned the penalty score of -10.000. The FBEs were normalized in two ways: correcting using an average calculated on a position-dependent basis (epithet: position-per-position; acronym: npp) or correcting using an average calculated over all positions (acronym: nap). Normalised FBEs were thus calculated using the following formula:

where FBE_i _is the binding energy of the i-th peptide,  is the average for a given position (npp) or over all positions (nap), FBE_max _and FBE_min _- the maximum and minimum FBEs, respectively, for a given position (npp) or for all positions (nap). Normalized FBEs were multiplied by (-1) before being entered into the quantitative matrices (QMs) for ease of presentation. Thus, the positive FBEs correspond to preferred amino acids, and negative FBEs to non-preferred residues. Three QMs were derived: one QMnpp and two QMnap (one for 9 mers and one for 13 mers).

### Test set

A test set of 457 peptides known to bind HLA-DP2 was collected from the Immune Epitope Database [34] (November 2010 release). The peptides were of different length and originated from 24 proteins. Each protein was represented as a set of overlapping nonamers and the binding score of each nonamer was calculated as a sum of the weights of all nine positions. Peptides originating from one protein were arranged in descending order according to their binding score; the top 5%, 10%, 15%, 20% and 25% were selected and compared to the known binders. If the nonamer sequence is included in the known binder sequence, the predicted peptide was considered as a true predicted binder. The ratio of all true predicted binders to all binders in the test set defined the *sensitivity *of prediction at the given cut-off. In the case of flanking residues, the procedure was the same but the parent proteins were represented as a set of overlapping 13 mers. The test set used in the present study is given as Additional file 1.

## Results

### Docking score-based quantitative matrices (DS-QMs) for nonamers

A library of 172 peptides (19 amino acids × 9 positions + 1 original ligand) was built and each docked separately into the HLA-DP2 rigid binding site. Two QMs (QMnpp and QMnap) were derived based on normalized FBE, according to the method described in Methods. The two QMs are given in Table 1 (DS-QMnpp) and Table 2 (DS-QMnap), respectively. A good correlation exists between the two QMs (r = 0.997).

**Table 1:** DS-QMnpp for HLA-DP2 binding prediction.

	*p1*	*p2*	*p3*	*p4*	*p5*	*p6*	*p7*	*p8*	*p9*
Ala	-0.101	0.068	0.040	0.000	0.049	-0.079	-0.089	**0.189**	**0.102**
Arg	0.036	-0.017	0.030	**0.411**	**0.445**	0.073	**0.379**	-0.031	-0.753
Asn	-0.046	0.011	-0.122	0.009	-0.116	0.090	-0.037	-0.007	**0.148**
Asp	-0.263	-0.071	-0.493	-0.237	-0.403	-0.458	-0.408	-0.287	-0.167
Cys	-0.065	0.002	-0.173	-0.076	-0.146	-0.076	-0.081	-0.027	**0.169**
Gln	0.027	-0.003	-0.163	0.053	-0.048	0.086	0.006	-0.139	**0.146**
Glu	-0.208	-0.045	-0.559	-0.169	-0.417	-0.383	-0.380	-0.379	-0.124
Gly	-0.202	0.004	-0.143	-0.035	0.016	-0.187	-0.150	**0.149**	0.007
His	**0.150**	**0.179**	0.015	0.002	-0.018	**0.235**	-0.055	-0.027	-0.132
Ile	**0.105**	0.085	-0.036	-0.006	-0.025	0.097	0.071	0.033	-0.050
Leu	**0.130**	0.056	**0.111**	**0.113**	-0.021	**0.107**	**0.167**	0.081	-0.044
Lys	-0.009	-0.071	-0.031	**0.428**	**0.597**	0.083	**0.592**	-0.139	**0.247**
Met	0.064	0.037	-0.102	0.002	-0.123	0.083	-0.007	-0.091	**0.187**
Phe	**0.469**	**0.101**	**0.269**	0.070	0.043	**0.542**	0.050	**0.177**	-10.000
Pro	-0.531	-0.762	**0.441**	-0.572	**0.394**	-10.000	-10.000	**0.621**	-10.000
Ser	-0.202	-0.019	-0.143	-0.090	-0.157	-0.194	-0.206	-0.071	0.074
Thr	-0.105	0.054	0.071	-0.077	-0.126	-0.153	-0.107	-0.043	**0.144**
Trp	**0.375**	**0.238**	**0.375**	0.084	0.093	-0.302	**0.238**	0.041	-10.000
Tyr	**0.324**	0.054	**0.395**	0.093	-0.038	**0.411**	0.056	-0.187	-10.000
Val	0.052	0.099	**0.218**	-0.003	0.002	0.026	-0.039	**0.137**	0.047

Binding scores are normalized position per position. Non-binding amino acids were assigned penalty score -10.000. Values above 0.100 are emboldened.

**Table 2:** DS-QMnap for HLA-DP2 binding prediction.

	*p1*	*p2*	*p3*	*p4*	*p5*	*p6*	*p7*	*p8*	*p9*
Ala	-0.248	0.092	0.059	0.054	0.015	-0.099	-0.007	0.007	**0.176**
Arg	-0.151	0.041	0.057	**0.430**	**0.178**	-0.037	**0.293**	-0.070	-0.425
Asn	-0.209	0.058	0.015	0.062	-0.053	-0.030	0.026	-0.062	**0.208**
Asp	-0.364	0.009	-0.087	-0.163	-0.172	-0.255	-0.212	-0.159	-0.013
Cys	-0.223	0.053	0.001	-0.016	-0.066	-0.098	-0.002	-0.069	**0.224**
Gln	-0.158	0.050	0.004	**0.103**	-0.025	-0.031	0.054	-0.108	**0.207**
Glu	-0.325	0.025	-0.105	-0.101	-0.177	-0.225	-0.194	-0.191	0.018
Gly	-0.321	0.054	0.009	0.022	0.001	-0.144	-0.046	-0.007	**0.110**
His	-0.070	**0.157**	0.053	0.055	-0.013	0.030	0.015	-0.069	0.012
Ile	-0.102	**0.101**	0.039	0.048	-0.016	-0.027	0.096	-0.048	0.069
Leu	-0.084	0.085	0.079	**0.157**	-0.014	-0.023	**0.157**	-0.031	0.073
Lys	-0.183	0.009	0.040	**0.445**	**0.241**	-0.032	**0.430**	-0.108	**0.278**
Met	-0.131	0.073	0.020	0.055	-0.056	-0.032	0.046	-0.091	**0.236**
Phe	**0.157**	**0.111**	**0.122**	**0.118**	0.012	**0.157**	0.082	0.002	-10.000
Pro	-0.555	-0.397	**0.169**	-0.470	**0.157**	-10.000	-10.000	**0.157**	-10.000
Ser	-0.321	0.040	0.009	-0.028	-0.070	-0.147	-0.083	-0.084	**0.157**
Thr	-0.251	0.083	0.068	-0.017	-0.058	-0.130	-0.019	-0.074	**0.206**
Trp	0.090	**0.192**	**0.151**	**0.130**	0.033	-0.191	**0.203**	-0.045	-10.000
Tyr	0.054	0.083	**0.157**	**0.139**	-0.021	**0.103**	0.086	-0.124	-10.000
Val	-0.140	**0.110**	**0.108**	0.051	-0.005	-0.056	0.025	-0.012	**0.137**

Binding scores are normalized over all positions. Non-binding amino acids were assigned penalty score -10.000. Values above 0.100 are emboldened.

According to QMnpp, the preferred amino acids at position 1 (p1) are Phe, Trp and Tyr, followed by His, Leu and Ile. QMnap selects only Phe, Trp and Tyr as preferred amino acids for p1. The X-ray structure shows that the p1 pocket is deep and hydrophobic [27]. It can accommodate all hydrophobic residues, including large aromatic amino acids, such as Phe, Trp and Tyr.

Peptide positions 2 and 3 (p2 and p3) project out from the binding site. Both QMs select Trp, His and Phe as preferred and Pro as non-preferred amino acid for p2. A great variety of other residues are well tolerated at p3, such as Pro, Tyr, Trp, Phe and Val; while Glu and Asp are disfavoured.

The binding pocket p4 is large, shallow and negatively charged due to Glu^26β^, Glu^68β ^and Glu^69β ^[27]. It strongly attracts positively charged amino acids as Arg and Lys, and distracts Asp and Glu. Leu, Tyr, Trp and Phe also are well accepted here, while Pro is not preferred.

Position 5 (p5) protrudes from the binding cleft but it is still in close proximity to the negatively charged residues Glu^26β^, Glu^68β ^and Glu^69β^. That explains the preferences for the positively charged Arg and Lys and the avoidance of Asp and Glu.

The binding pocket p6 is deep and hydrophobic like pocket p1 [27]. Phe, Tyr and His are well accepted here; Pro does not bind at all; while Asp, Glu and Trp are deleterious.

Position 7 (p7) lies tangentially to the binding site and is considered as a secondary anchor position for some MHC class II proteins [20,35]. It is also in the vicinity of Glu^26β^, Glu^68β ^and Glu^69β ^and prefers Lys and Arg but avoids Asp and Glu. Trp also binds well here, while Pro does not bind at all.

Position 8 (p8) is solvent-exposed, yet shows a strong preference for Pro. Although it is far from Glu^26β^, Glu^68β ^and Glu^69β^, their influence on binding preferences is clear. Glu and Asp are deleterious at p8.

The binding pocket 9 (p9) accepts large aliphatic, polar, or even charged residues [27]. Accordingly, there is a wide variety of preferred amino acids at this position: Lys, Met, Cys, Gln, Asn, and Thr. In contrast, Pro and large aromatic amino acids, such as Phe, Trp and Tyr, do not bind at all; nor is Arg tolerated here.

### External validation

**Figure 1:**
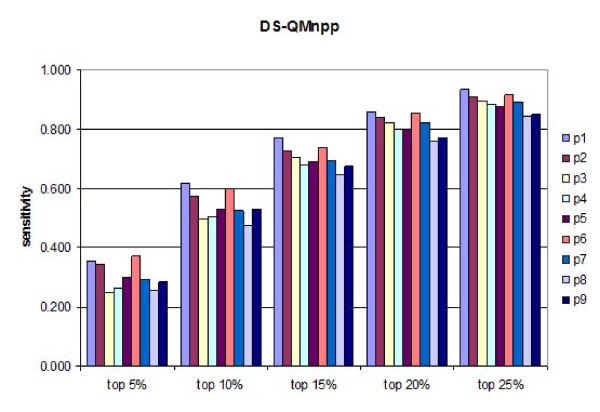
**Sensitivities of the predictions calculated at five different thresholds (5%, 10%, 15%, 20% and 25%) for each peptide binding core position by DS-QMnpp**.

**Figure 2:**
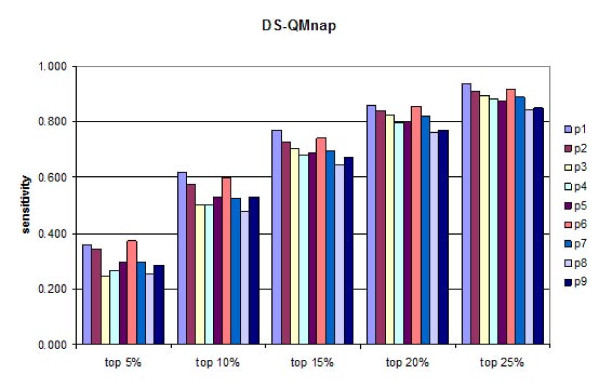
**Sensitivities of the predictions calculated at five different thresholds (5%, 10%, 15%, 20% and 25%) for each peptide binding core position by DS-QMnap**.

**Figure 3:**
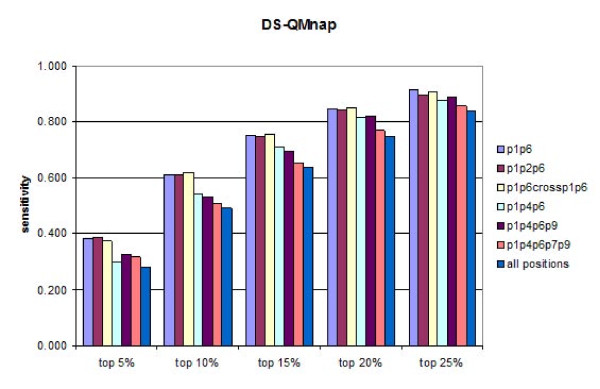
**Sensitivities of the predictions calculated at five different thresholds (5%, 10%, 15%, 20% and 25%) for different combinations of peptide binding core positions by DS-QMnap**.

A test set of 457 peptides known to bind HLA-DP2 originating from 24 proteins was used for external validation of the derived DS-QMs. Initially, the predictive ability of every position was assessed. Subsequently, different combinations of positions were evaluated. The sensitivities of the predictions were calculated at five different thresholds (5%, 10%, 15%, 20% and 25%) for each position are given at Figures 1 and 2. It is evident that QMnap and QMnpp predict equally well. QMnap was used next in the study. The highest predictive ability belongs to p6, followed by p1 and p2. The best two-position and three-position combinations slightly improve the predictions (Figure 3, models "p1p6" and "p1p2p6"). Addition of a cross term between p1 and p6 has no impact on the predictions (Figure 3, model "p1p6crossp1p6"). Combinations between anchor positions were also tested (Figure 3, models "p1p4p6p9" and "p1p4p6p7p9"). No improvement was seen. The combination of all positions also shows a lower predictive ability than the "p1p6" model, thus considering for the non-additivity of binding (Figure 3, model "all positions"). If the contribution made by each pocket to the overall binding energy was formally additive, then the model containing all pocket residues would have had the highest sensitivity. This was not the case.

### Comparison to existing servers for HLA-DP2 binding prediction

**Figure 4:**
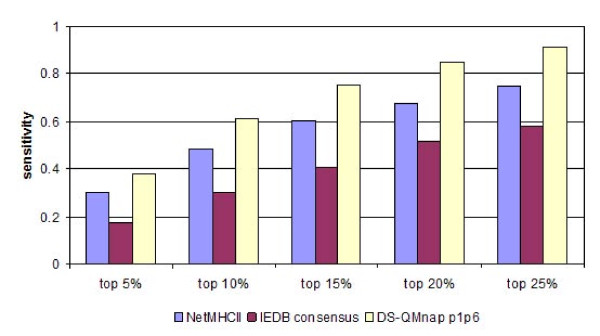
**Sensitivities of the predictions calculated at five different thresholds (5%, 10%, 15%, 20% and 25%) by different servers for DP2 binding prediction**.

To the best of our knowledge, only three other servers exist for peptide HLA-DP2 binding prediction: NetMHCII [36], IEDB [37] and MultiRTA [38]. All three are sequence-based methods. NetMHCII and IEDB use artificial neural networks, while MultiRTA applies the Regularized Thermodynamic Average (RTA) prediction method [39]. However, MultiRTA selects only one binder from a protein, and it is not suitable for use with our test set, which consists of many binders originating from a limited number of proteins. Thus, the comparative study only includes NetMHCII, IEDB, and the DS-QMnap model. The 24 proteins from the test set were cleaved into successive overlapping nonamers. Sensitivities at different cutoffs were recorded (Figure 4). It is evident that DS-QMnap performed best when compared to the existing servers for DP2 binding prediction. It predicts 38% of the true binders at the top 5% threshold, 61% at top 10%, 75% at top 15%, 85% at top 20%, and 92% at top 25%.

### Effect of the flanking residues on the peptide binding affinities

In the present study, we also examined the influence of flanking residues on peptide binding affinities. Four flanking residues were considered: two at each end. Seventy six additional peptides (19 amino acids × 4 positions) were modelled and docked into HLA-DP2 and the FBEs derived from the docking experiments were normalized using either a position-dependent average (npp) or an overall average taken over all positions (nap). Similarly to the evaluation of nonamers, two QMs were derived: QMnpp and QMnap. They are given in Additional file 2. The two are highly correlated (r = 0.995), thus only QMnap was chosen to test predictivity.

The preferred amino acids at p-1 (the first before p1) were Lys, Arg and Pro, while non-preferred residues were Asp and Glu. This preference could be explained by the presence of Glu^55α ^in close proximity to p-1. Positon p-2 (the second before p1) can accommodate a great variety of amino acids, including Pro, Trp, Ala, Arg, Gly, Lys and Phe. No disfavoured amino acids were seen for this position. Thr, Phe, Cys, Ile and Val are well accepted at p+1 (the first after p9), Pro is deleterious. Finally, Gly, Pro and Trp are accommodated well at position p+2 (the second flanking position after p9), while Thr is not favoured here.

**Figure 5:**
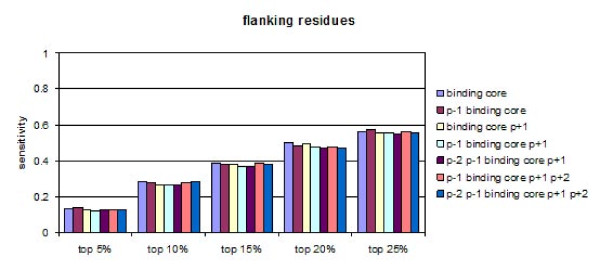
**Sensitivities of the predictions calculated at five different thresholds (5%, 10%, 15%, 20% and 25%) for different combinations of peptide binding core positions and flanking residues by DS-QMnap**.

The proteins from the test set were also converted to sets of overlapping 13 mers. The binding score of each 13 mer was calculated using the 13 mer-specific QMnap. Note that using overlapping 13 mers significantly decreases the sensitivity, since the number of distinct registers originating from one binder decreases. Several combinations of flanking residues were compared. The first bars in Figure 5 give the sensitivities calculated when only the binding core of nine amino acids was considered (the centre of each 13 mer); subsequent bars show the sensitivities for different combinations of binding core and flanking residues. It is evident that the addition of flanking residues does not improve the predictions.

### Identification of the peptide binding core

The binding peptide RKFHYLPFLPSTGGS from the X-ray structure of the peptide - HLA-DP2 protein complex was used to test if the molecular docking procedure could identify the peptide binding core. The binding 15 mer was presented as a set of overlapping peptides, with a moving binding core shown in bold in Table 3. The FBEs of the peptides and their binding scores calculated by the best DS-QMnap model "p1p6", are given in Table 3. It is evident that both methods clearly discriminated the binding core, since derived scores were significantly higher than the scores derived from the rest of the overlapping peptides.

**Table 3:** Identification of peptide binding core by molecular docking and DS-QM.

*Peptide bound* *to HLA-DP2*	*Binding mode* *Binding core is given in bold*	*AutoDock* *FBE (kcal/mol)*	*DS-QMnap p1p6* *binding score*
pdb code: 3lqz	**RKFHYLPFL**PSTGGS	9.43	-0.174
	R**KFHYLPFLP**STGGS	8.53	-10.183
Binding core	RK**FHYLPFLPS**TGGS	**-5.36**	**0.314**
	RKF**HYLPFLPST**GGS	6.36	-0.093
	RKFH**YLPFLPSTG**GS	6.69	-9.043
	RKFHY**LPFLPSTGG**S	3.06	-0.231
	RKFHYL**PFLPSTGGS**	3.72	-0.685
			
Apolipoprotein A-II 12-27	**LQSLVSQYF**QTVADYA	0.26	-0.231
	L**QSLVSQYFQ**TVADYA	-0.5	-0.189
	LQ**SLVSQYFQT**VADYA	-0.36	-0.218
Binding core	LQS**LVSQYFQTV**ADYA	4.54	**0.073**
	LQSL**VSQYFQTVA**DYA	-1.75	-0.171
	LQSLV**SQYFQTVAD**YA	2.48	-0.451
			
Cathepsin S 182-197	**GGFMTTAFQ**YIIDNKG	-0.09	-0.451
	G**GFMTTAFQY**IIDNKG	0.26	-0.42
Binding core	GG**FMTTAFQYI**IDNKG	**-5.99**	**0.314**
	GGF**MTTAFQYII**DNKG	-2.91	-0.162
	GGFM**TTAFQYIID**NKG	3.79	-0.148
	GGFMT**TAFQYIIDN**KG	3.04	-0.278
	GGFMTT**AFQYIIDNK**G	1.03	-0.275
	GGFMTTA**FQYIIDNKG**	-4.91	-0.098
			
Igλ 188-204	**QSNNKYAAS**SYLSLTPE	1.42	-0.055
	Q**SNNKYAASS**YLSLTPE	-1.86	-0.42
	QS**NNKYAASSY**LSLTPE	0.4	-0.308
	QSN**NKYAASSYL**SLTPE	-0.15	-0.356
	QSNN**KYAASSYLS**LTPE	-0.87	-0.33
Binding core	QSNNK**YAASSYLSL**TPE	**-5.06**	**0.157**
	QSNNKY**AASSYLSLT**PE	-0.3	-0.271
	QSNNKYA**ASSYLSLTP**E	-2.81	-0.395
	QSNNKYAA**SSYLSLTPE**	0.09	-0.344
			
Interferon-induced protein 1-8D 53-65	**VPDHVVWSL**FNTL	-4.92	-0.196
	V**PDHVVWSLF**NTL	-1.81	-0.746
	VP**DHVVWSLFN**TL	-3.19	-0.511
	VPD**HVVWSLFNT**L	-3.28	-0.093
Binding core	VPDH**VVWSLFNTL**	**-6.19**	**0.017**
			
LR11 mosaic ptorein	**VYGIFYATS**FLDLYRNP	-2.48	-0.037
	V**YGIFYATSF**LDLYRNP	-1.49	-0.045
	VY**GIFYATSFL**DLYRNP	-3.97	-0.451
	VYG**IFYATSFLD**LYRNP	-1.38	-0.249
Binding core	VYGI**FYATSFLDL**YRNP	**-5.98**	**0.314**
	VYGIF**YATSFLDLY**RNP	2.11	0.031
	VYGIFY**ATSFLDLYR**NP	7.07	-0.503
	VYGIFYA**TSFLDLYRN**P	2.12	-0.274
	VYGIFYAT**SFLDLYRNP**	-3.66	-0.218

The FBE values and the binding scores of the identified binding cores are given in bold.

The same procedure was applied to five additional known DP2 binders [40] (Table 3). The FBE values identified four of the five binding cores, while the DS-QMnap model found all the five cores.

## Discussion

Molecular docking is a key structure-based method of immunoinformatics. In contrast to sequence-based methods, docking experiments do not require extensive pre-existing experimental data. The only information necessary is an X-ray structure of the peptide - MHC protein complex. Recently, the docking methodology was extensively tested on both peptide - MHC class I and peptide - MHC class II complexes: it proved to be a rapid and accurate method for evaluating peptide binding to MHCs [41].

Although the structures of a number of HLA-DR and HLA-DQ alleles have long been available [20,35], the structure of a HLA-DP protein was only solved recently [27]. This has now enabled us to apply structure-based molecular docking to the analysis of the interaction interface of the HLA-DP2 peptide complex. The X-ray structure of the binding peptide was used as a starting template to create a combinatorial library of 247 peptides built using the SAAS principle. Using this, we were able to explore the structure-activity relationships of the nine binding core positions and the four flanking positions, two on each end. Peptides were docked into the DP2 binding site using AutoDock. The lowest resulting FBEs were recorded, normalized, and used to create DS-QMs. The predictive ability of these QMs was tested using an external test set and compared to existing servers for DP2 binding prediction. A similar docking-based procedure was applied recently to 12 HLA-DR1 proteins indicating that DS-QMs have good predictive ability [42].

Analysis of DS-QMs coupled to the external predictivity tests lead to several clear conclusions. The anchor positions p1 and p6 take a leading role in the binding predictions. Hydrophobic aromatic amino acids, like Phe, Tyr and Trp, are preferred at these two positions. Thus, our results confirm the unique binding motif for DP2 [43] and other DP alleles [44]. The prediction tests show that p1+p6 with or without a cross term p1p6 are self-sufficient to identify 38% of the true binders among the top 5% of the best predicted peptides, 61% among the top 10%, and 75% among the top 15% (Figure 3).

The anchors p4 and p9 have a low impact on the peptide binding prediction when used either as single predictors or in combination (Figures 1, 2 and 3). Instead, p2 is found to be the third most important position after p6 and p1 (Figures 1 and 2). It works equally well as a single predictor and in combination with p1 and p6 (Figure 3). Aromatic amino acids are preferred here, such as Trp, His and Phe. These preferences could be explained by the presence of residue His^79β^, situated close to the side chain of p2, thus enabling the stacking of aromatic rings [45].

The most striking feature of the peptide - HLA-DP2 complex is the unique solvent exposed acidic pocket formed between the bound peptide backbone and the protein α-helix. It contains three glutamic acids: Glu^26β^, Glu^68β ^and Glu^69β^. Additionally, close to this acidic triad there is another glutamic acid: Glu^67β^. The strong negative electrostatic potential created by the four nominally negatively-charged residues, determines the amino acid preferences within the main part of the binding core. All six positions between positions p3 and p8 disfavour Glu and Asp. Positions p4, p5 and p7 prefer Lys and Arg. It has been hypothesized that this acidic pocket is able to bind divalent inorganic cations (e.g., Ca^2+^, Mg^2+^, Co^2+^, Be^2+^, etc.); this forms an explanation for the association that DP2 has with hard metal lung disease [27].

Analysis of amino acid preferences for all nine binding core positions reveals the ambiguous role of Pro. Pro is a preferred amino acid at peptide positions p3, p5 and p8 yet is not-preferred at p1, p2 and p4. When Pro is present at positions p6, p7 and p9, peptides do not bind at all. As Pro does not possess an interactive side chain, its role in the peptide binding is connected mainly with restrictions to the backbone conformation. This highlights the complex and conflicting influences at play here. Certain deep, hydrophobic inward-facing pockets seem highly selective, while the more exposed pockets tolerate more and more variable residue types. This is to be expected. No binding data exists for the TCR recognition of the HLA-DP2 pMHC complex, which would indicate the steric and physic-chemical constraints exerted on immunologically-active epitopes as opposed to binding peptides.

No effect of the flanking residues was found on the peptide binding predictions to DP2, although all four of them show strong preferences for particular amino acids. Pro also plays an ambiguous role here, being preferred at positions p-2, p-1 and p+2 and non-preferred at position p+1.

Additionally, the DS-QMs were used to identify the binding core of six known DP2 binding peptides, one of them taken from the X-ray structure. All six binding cores were identified with scores significantly higher than the scores derived for the rest of the overlapping peptides.

The DS-QMs derived in the present study were compared to experimental studies based on SAAS peptides binding to HLA-DP2. Berretta *et al*. [46] performed competition tests with the Ii-derived peptide CLIP and its SAAS peptides in p4 and p6 binding to DP2. Pocket 4 showed high affinity for positively charged, aromatic, and polar residues, whereas aliphatic residues were disfavoured. Pocket 6 showed high affinity for aromatic residues. Both experimentally-determined pocket preferences agree in full with our DS-QMs. Sidney et al. [44] also performed a SAAS analysis of the binding specificities of HLA-DPB1*0201. They defined a binding motif for DP2 including preferred amino acids at peptide positions p-2 (Ala, Phe, Lys, Ser, Thr, Val, Trp, Tyr), p1 (Phe, Ile, Leu, Met, Val, Trp, Tyr) and p6 (Phe, Ile,Leu, Met, Trp, Tyr). The DS-QMs are in partial agreement with these preferences. According to the DS-QMs, Ser, Thr and Tyr are not among the preferred amino acids at p-2, and Trp is not accepted at p6. Most recently, Greenbaum et al. [47] found a high degree of overlapping repertoire amongst all HLA class II molecules due to binding of multiple registers and dominant backbone interactions than peptide anchor preferences.

We can say with some confidence that both molecular docking procedure and the DS-QM based peptide binding prediction identify the binding core of the bound peptide from the X-ray structure in a straightforward manner [27]. Moreover, the comparison to other servers suggests that the method described in the present study should provide a reliable tool for DP2 binding prediction.

## Conclusion

Amongst immunoinformatics problems, the prediction of class II peptide-MHC binding has recently been the subject of much critical comment [15-17]. We must set this against the background of prediction in general, which is still treated with considerable scepticism by many. In many ways, accurate quantitative and qualitative prediction is the ultimate goal of scientific endeavour, since it affords us both true certainty in our understanding and also greatly augmented abilities to manipulate and design. The present study has continued our exploration of docking as an approach to the difficult and challenging problem of class II MHC-peptide binding prediction. In future work, we will explore more complete docking protocols that allow energetic relaxation of both the peptide and the protein, and also make use of a wider range of different scoring functions; as well as extending our analysis to include a wider range of class II alleles and undertaking prospective as well as retrospective analysis.

## Abbreviations

QM: quantitative matrix; DS-QM: docking score-based quantitative matrix; SAAS: single amino acid substitution; FBE: free binding energy.

## Authors' contributions

IrDo designed the study, modelled the input structures and drafted the manuscript. AP performed the molecular dockings. IvDi performed the external validation. DRF advised on the study and helped with the writing of the manuscript. All authors revised and approved its final version.

## Supplementary Material

Additional file 1 **Test set of known HLA-DP2 binders**. The file contains a test set of 457 known peptide binders to HLA-DP2, parent protein NCBI GI numbers and IC50 values. Click here for file

Additional file 2 **DS-QMnpp for HLA-DP2 binding prediction of 13 mer peptides**. The file contains the DS-QMnpp for HLA-DP2 binding prediction of 13 mer peptides. Binding scores are normalized position per position. Non-binding amino acids were assigned a binding score of -10.000. Click here for file
