# Supplementary material for: Peptide binding prediction for the human class II MHC allele HLA-DP2: a molecular docking approach
Source: BMC Struct Biol. 2011 Jul 14;11:32. doi: 10.1186/1472-6807-11-32 (PMC3146810; doi:10.1186/1472-6807-11-32)
Supplement: Additional file 2 — DS-QMnpp for HLA-DP2 binding prediction of 13 mer peptides. The file contains the DS-QMnpp for HLA-DP2 binding prediction of 13 mer peptides. Binding scores are normalized position per position. Non-binding amino acids were assigned a binding score of -10.000. [file 1472-6807-11-32-S2.DOC]

QM for HLA-DP2 binding prediction of 13mer peptides. Binding score are normalized position per position. Non-binding amino acids were assigned binding score -10.000.

|  | p-2 | p-1 | p1 | p2 | p3 | p4 | p5 | p6 | p7 | p8 | p9 | p+1 | p+2 |
| --- | --- | --- | --- | --- | --- | --- | --- | --- | --- | --- | --- | --- | --- |
| Ala | 0.213 | 0.080 | -0.101 | 0.068 | 0.040 | 0.000 | 0.049 | -0.079 | -0.089 | 0.189 | 0.102 | 0.158 | 0.150 |
| Arg | 0.219 | 0.391 | 0.036 | -0.017 | 0.030 | 0.411 | 0.445 | 0.073 | 0.379 | -0.031 | -0.753 | -0.076 | -0.071 |
| Asn | -0.118 | 0.001 | -0.046 | 0.011 | -0.122 | 0.009 | -0.116 | 0.090 | -0.037 | -0.007 | 0.148 | 0.105 | -0.097 |
| Asp | -0.394 | -0.603 | -0.263 | -0.071 | -0.493 | -0.237 | -0.403 | -0.458 | -0.408 | -0.287 | -0.167 | -0.339 | -0.280 |
| Cys | -0.113 | -0.084 | -0.065 | 0.002 | -0.173 | -0.076 | -0.146 | -0.076 | -0.081 | -0.027 | 0.169 | 0.251 | -0.092 |
| Gln | -0.063 | -0.236 | 0.027 | -0.003 | -0.163 | 0.053 | -0.048 | 0.086 | 0.006 | -0.139 | 0.146 | 0.139 | -0.007 |
| Glu | -0.571 | -0.671 | -0.208 | -0.045 | -0.559 | -0.169 | -0.417 | -0.383 | -0.380 | -0.379 | -0.124 | -0.403 | -0.033 |
| Gly | 0.186 | -0.044 | -0.202 | 0.004 | -0.143 | -0.035 | 0.016 | -0.187 | -0.150 | 0.149 | 0.007 | -0.027 | 0.376 |
| His | -0.052 | -0.084 | 0.150 | 0.179 | 0.015 | 0.002 | -0.018 | 0.235 | -0.055 | -0.027 | -0.132 | 0.114 | -0.067 |
| Ile | 0.020 | 0.142 | 0.105 | 0.085 | -0.036 | -0.006 | -0.025 | 0.097 | 0.071 | 0.033 | -0.050 | 0.207 | 0.023 |
| Leu | 0.009 | 0.108 | 0.130 | 0.056 | 0.111 | 0.113 | -0.021 | 0.107 | 0.167 | 0.081 | -0.044 | 0.110 | 0.014 |
| Lys | 0.186 | 0.476 | -0.009 | -0.071 | -0.031 | 0.428 | 0.597 | 0.083 | 0.592 | -0.139 | 0.247 | -0.047 | -0.254 |
| Met | -0.102 | 0.024 | 0.064 | 0.037 | -0.102 | 0.002 | -0.123 | 0.083 | -0.007 | -0.091 | 0.187 | 0.070 | 0.018 |
| Phe | 0.108 | 0.029 | 0.469 | 0.101 | 0.269 | 0.070 | 0.043 | 0.542 | 0.050 | 0.177 | -10.000 | 0.261 | 0.052 |
| Pro | 0.429 | 0.397 | -0.531 | -0.762 | 0.441 | -0.572 | 0.394 | -10.000 | -10.000 | 0.621 | -10.000 | -0.612 | 0.367 |
| Ser | -0.135 | -0.050 | -0.202 | -0.019 | -0.143 | -0.090 | -0.157 | -0.194 | -0.206 | -0.071 | 0.074 | 0.012 | 0.091 |
| Thr | -0.102 | -0.005 | -0.105 | 0.054 | 0.071 | -0.077 | -0.126 | -0.153 | -0.107 | -0.043 | 0.144 | 0.339 | -0.624 |
| Trp | 0.274 | 0.069 | 0.375 | 0.238 | 0.375 | 0.084 | 0.093 | -0.302 | 0.238 | 0.041 | -10.000 | 0.192 | 0.320 |
| Tyr | -0.091 | -0.146 | 0.324 | 0.054 | 0.395 | 0.093 | -0.038 | 0.411 | 0.056 | -0.187 | -10.000 | -0.661 | 0.010 |
| Val | 0.097 | 0.205 | 0.052 | 0.099 | 0.218 | -0.003 | 0.002 | 0.026 | -0.039 | 0.137 | 0.047 | 0.207 | 0.103 |

QM for HLA-DP2 binding prediction of 13mer peptides. Binding score are normalized over all positions. Non-binding amino acids were assigned binding score -10.000.

|  | p-2 | p-1 | p1 | p2 | p3 | p4 | p5 | p6 | p7 | p8 | p9 | p+1 | p+2 |
| --- | --- | --- | --- | --- | --- | --- | --- | --- | --- | --- | --- | --- | --- |
| Ala | 0.137 | 0.041 | -0.266 | 0.073 | 0.041 | 0.036 | -0.003 | -0.117 | -0.026 | -0.012 | 0.158 | 0.087 | 0.065 |
| Arg | 0.139 | 0.118 | -0.169 | 0.023 | 0.039 | 0.412 | 0.160 | -0.055 | 0.275 | -0.088 | -0.443 | 0.020 | -0.007 |
| Asn | 0.054 | 0.022 | -0.227 | 0.040 | -0.003 | 0.044 | -0.071 | -0.048 | 0.008 | -0.080 | 0.190 | 0.072 | -0.016 |
| Asp | -0.016 | -0.127 | -0.382 | -0.009 | -0.105 | -0.182 | -0.190 | -0.273 | -0.230 | -0.177 | -0.031 | -0.055 | -0.076 |
| Cys | 0.055 | 0.001 | -0.241 | 0.034 | -0.017 | -0.034 | -0.084 | -0.116 | -0.020 | -0.087 | 0.206 | 0.114 | -0.014 |
| Gln | 0.068 | -0.037 | -0.176 | 0.032 | -0.014 | 0.085 | -0.044 | -0.049 | 0.036 | -0.126 | 0.189 | 0.082 | 0.013 |
| Glu | -0.060 | -0.144 | -0.343 | 0.007 | -0.123 | -0.119 | -0.195 | -0.243 | -0.212 | -0.209 | 0.000 | -0.073 | 0.005 |
| Gly | 0.130 | 0.011 | -0.339 | 0.036 | -0.009 | 0.004 | -0.017 | -0.162 | -0.065 | -0.026 | 0.091 | 0.034 | 0.139 |
| His | 0.071 | 0.001 | -0.088 | 0.139 | 0.034 | 0.037 | -0.031 | 0.012 | -0.003 | -0.087 | -0.006 | 0.075 | -0.006 |
| Ile | 0.089 | 0.057 | -0.120 | 0.083 | 0.020 | 0.030 | -0.034 | -0.045 | 0.078 | -0.066 | 0.051 | 0.101 | 0.023 |
| Leu | 0.086 | 0.048 | -0.102 | 0.066 | 0.061 | 0.139 | -0.032 | -0.041 | 0.139 | -0.049 | 0.055 | 0.073 | 0.020 |
| Lys | 0.130 | 0.139 | -0.201 | -0.009 | 0.022 | 0.427 | 0.222 | -0.051 | 0.412 | -0.126 | 0.260 | 0.029 | -0.067 |
| Met | 0.058 | 0.027 | -0.149 | 0.055 | 0.002 | 0.037 | -0.074 | -0.051 | 0.027 | -0.109 | 0.218 | 0.062 | 0.022 |
| Phe | 0.111 | 0.029 | 0.139 | 0.093 | 0.104 | 0.100 | -0.006 | 0.139 | 0.064 | -0.016 | -10.000 | 0.117 | 0.033 |
| Pro | 0.192 | 0.119 | -0.573 | -0.415 | 0.151 | -0.488 | 0.139 | -10.000 | -10.000 | 0.139 | -10.000 | -0.133 | 0.136 |
| Ser | 0.050 | 0.009 | -0.339 | 0.022 | -0.009 | -0.046 | -0.088 | -0.165 | -0.101 | -0.102 | 0.139 | 0.046 | 0.046 |
| Thr | 0.058 | 0.020 | -0.269 | 0.065 | 0.050 | -0.035 | -0.076 | -0.148 | -0.037 | -0.092 | 0.188 | 0.139 | -0.188 |
| Trp | 0.153 | 0.039 | 0.072 | 0.174 | 0.133 | 0.112 | 0.015 | -0.209 | 0.185 | -0.063 | -10.000 | 0.097 | 0.121 |
| Tyr | 0.061 | -0.014 | 0.036 | 0.065 | 0.139 | 0.121 | -0.039 | 0.085 | 0.068 | -0.143 | -10.000 | -0.147 | 0.019 |
| Val | 0.108 | 0.072 | -0.158 | 0.091 | 0.090 | 0.033 | -0.023 | -0.074 | 0.007 | -0.030 | 0.119 | 0.101 | 0.050 |
